# Impact of imaging cross-section on visualization of thyroid microvessels using ultrasound: Pilot study

**DOI:** 10.1038/s41598-019-57330-w

**Published:** 2020-01-15

**Authors:** Rohit Nayak, Noshin Nawar, Jeremy Webb, Mostafa Fatemi, Azra Alizad

**Affiliations:** 1 Department of Radiology, Mayo Clinic College of Medicine and Science, Rochester, Minnesota 55905 United States; 2 Department of Physiology and Biomedical Engineering, Mayo Clinic College of Medicine and Science, Rochester, Minnesota 55905 United States

**Keywords:** Cancer imaging, Translational research, Biomedical engineering

## Abstract

Non-invasive, contrast-free microvascular imaging of human thyroids can be potentially beneficial in reducing the large number of benign biopsies of suspicious nodules. However, motion incurred by thyroid due to its proximity to the pulsating carotid artery significantly impacts the visualization of blood flow in small vessels. Singular value based spatiotemporal clutter filtering (SVD-STF) improves the performance of tissue rejection in the presence of motion. However, despite effective clutter filtering, motion in thyroid imaging can impact coherent integration of the Doppler ensemble and degrade the visualization of the underlying vasculature. Recently studies have demonstrated that motion correction using 2D normalized cross-correlation based speckle tracking can address this issue, however, only in-plane motion can be tracked and corrected. Given the natural anatomical orientation of the rigid trachea, thyroid and the pulsating carotid artery, we hypothesize that imaging of thyroid microvessels may be more reliable in the longitudinal view than in the transverse. Specifically, distal presence of rigid trachea can limit out-of-plane motion in the longitudinal view. We tested this hypothesis on 48 acquisitions obtained from 24 thyroid patients having at least one suspicious nodule. In each patient, ultrasound images of the thyroid were acquired in both longitudinal and transverse views. Compounded plane-wave imaging was used to acquire the ultrasound images at high frame-rate, which is important for contrast-free small vessel blood flow imaging. Thyroid motion was tracked using 2D normalized cross-correlation based speckle tracking. Tissue clutter was rejected using singular value decomposition based spatiotemporal clutter filtering. The clutter-filtered Doppler ensemble was motion corrected prior to slow-time power Doppler integration. Signal-to-noise and contrast-to-noise ratios were computed to assess the improvement in quality of the power Doppler images. Out-of-plane motion was detected by estimating normalized ensemble cross-correlation coefficient. The results demonstrated that motion associated with the thyroid due to the carotid artery was primarily in the lateral direction, which could be estimated and corrected using 2D speckle tracking. However, the motion in the transverse view displayed increased speckle decorrelation. The average ensemble cross-correlation coefficient of the thyroid ultrasound images were significantly higher (p < 0.05) in the longitudinal view than in the transverse view. The largest improvement in SNR and CNR of the estimated PD images upon motion correction was observed in the longitudinal view (12.95 ± 3.76 dB and 16.48 ± 4.6 dB) than in the transverse view (3.72 ± 0.894 dB and 6.217 ± 1.689 dB). These preliminary results show that motion encountered by the thyroid due to carotid pulsations can be effectively tracked and corrected in the longitudinal view relative to transverse, which is important for reliably visualizing the underlying blood flow.

## Introduction

Non-invasive microvascular blood flow imaging can be acutely helpful in detection and diagnosis of thyroid cancer. Invasive fine needle aspiration biopsies of thyroid nodules – a majority of which are benign – is a considerable burden on the financial and health care system, and a source of distress in patients. Low specificity of sonographic features limits the scope of ultrasound imaging in non-invasively diagnosing thyroid cancer^1^.

**Figure 1:**
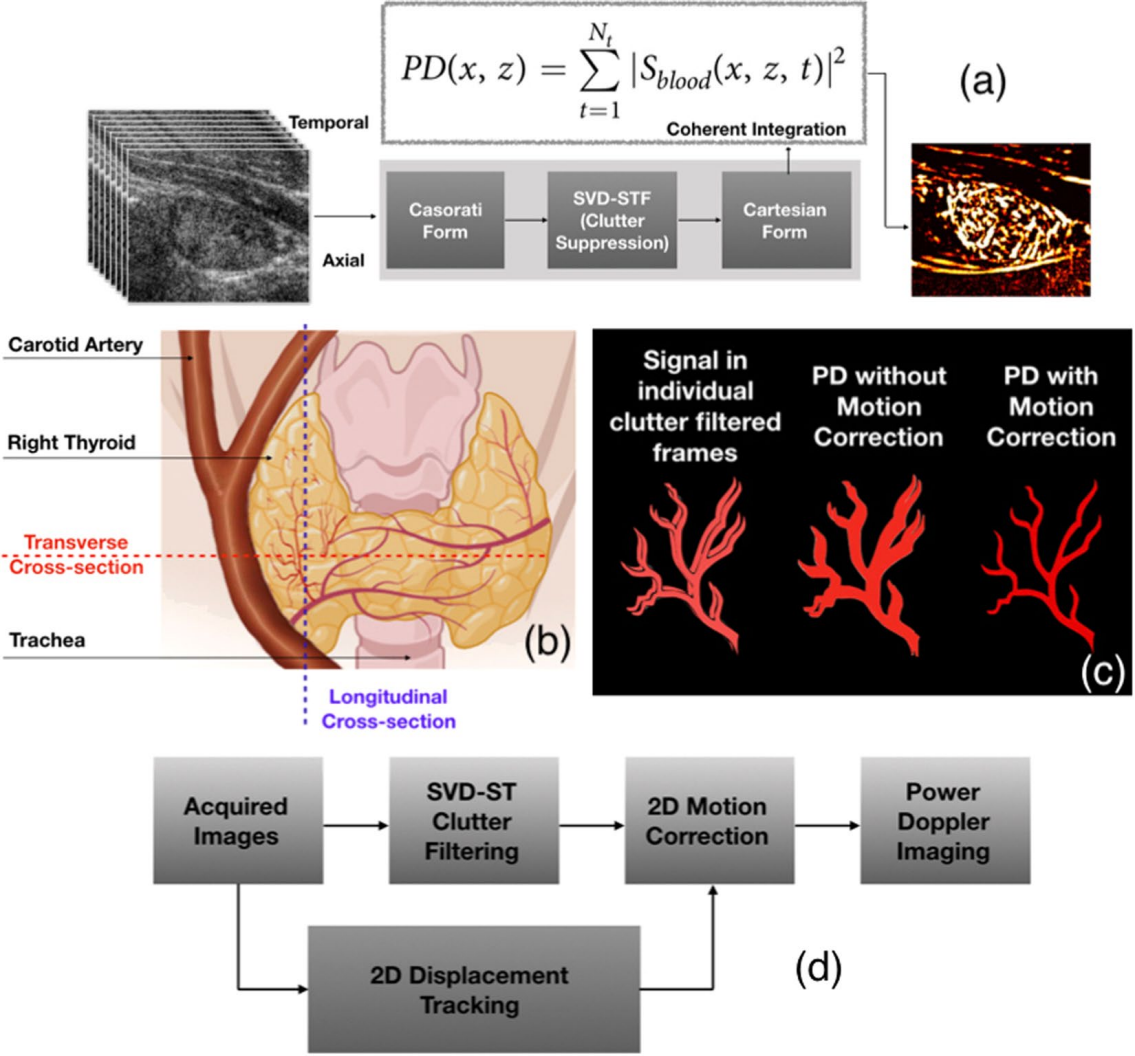
An illustrative example of impact of motion on blood flow visualization in thyroid imaging, across transverse and longitudinal views. (**a**) Overviews the steps involved in conventional SVD based spatiotemproal clutter-filtered power Doppler imaging involving a large ensemble of ultrasound images obtained at high frame-rate. (**b**) Displays the anatomical position of the thyroid gland, the pulsating carotid artery and the rigid trachea, with respect to the longitudinal and transverse planes. The image in (**b**) was created with BioRender.com (**c**) illustrates the impact of motion on coherent integration of the Doppler ensemble, and its potential impact on the visualization of the blood flow signal^14^. (**d**) Outlines the steps involved in correcting in-plane motion, prior to Doppler integration for improved visualization, as demonstrated in^14^.

**Figure 2:**
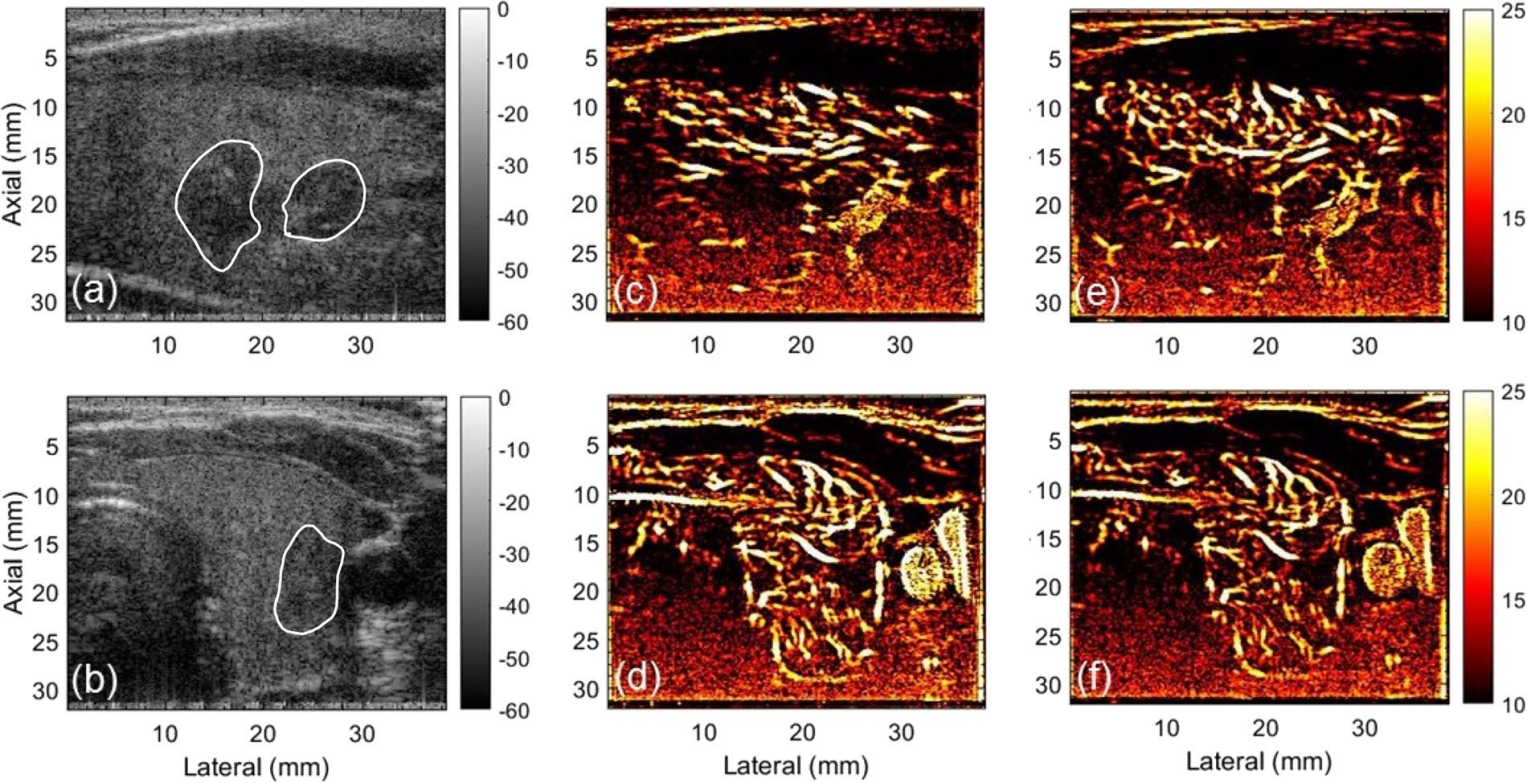
Displays the plane wave B-mode sonograms (**a**,**b**) and the corresponding PD images – without (**c**,**d**) and with (**e**,**f**) motion correction. (**a**,**c**,**e**) corresponds to the longitudinal plane, and (**b**,**d**,**f**) corresponds to transverse plane. For the sake of clarity in visualizing the small blood vessel structures, the tumor outline is indicated on the B-mode sonogram.

Assessment of thyroid malignancy based on intranodular and peripheral vascularity detected using Doppler ultrasound has been attempted by several researchers^2–11^. However, large tissue motion in unavoidable in thyroid imaging due to its proximity to the pulsating carotid artery – a major challenge in reliably imaging small vessel blood flow. Specifically, motion presents two major challenges: (1) complicates tissue clutter suppression [Fig. 1(a,b)], and (2) prevents coherent integration of the Doppler ensemble [Fig. 1(c)]. Recent developments in ultrafast imaging and clutter-filtering based on spatio-temporal coherence has significantly reduced the impact of motion on tissue clutter suppression^12,13^. Tissue components in an ultrafast acquisition typically corresponds to high spatio-temporal coherence (STC), compared to the blood flow signal, thus they can be effectively separated using singular value decomposition (SVD)^12,13^. To address the second issue of incoherent Doppler integration, we demonstrated that motion correction could substantially improve the performance of Doppler imaging of thyroid blood vessels [Fig. 1(c,d)]^14^. Specifically, thyroid motion was estimated as components of axial and lateral displacements using 2D normalized cross-correlation based speckle tracking of the Doppler ensemble, prior to clutter filtering. The estimated motion was used for rigid body based motion correction of the clutter-filtered Doppler ensemble^14^ to achieve coherent Doppler integration. However, reliable estimation of thyroid displacements is only feasible for in-plane motion, and presence of out-of-plane elevational motion can limit the scope of motion correction.

Thyroid scans are typically performed in longitudinal and transverse views [Fig. 1(b)]. However, motion characteristics of the thyroid in either views can be distinctly different due to its relative location with respect to the rigid trachea and the pulsating carotid artery^1,3,15^. Specifically in the longitudinal view, distal presence of trachea limits elevational motion, however at the expense of increased translational motion in the lateral direction^16,17^. Comparatively, in the transverse view, carotid induced thyroid motion – predominantly in the lateral direction – is restricted by the rigid trachea, which reciprocally translates in to elevational and axial expansions^18–20^ assuming tissue incompressibility. Difference in thyroid motion characteristics across the longitudinal and traverse planes have been investigated by several researchers^21^. World Federation of Ultrasound in Medicine and Biology (WFUMB) guidelines on clinical ultrasound strain elastography recommends the longitudinal view since its less susceptible to elevational motion^22^. However, in elastographic studies using carotid pulsations as the source of mechanical excitation, transverse view may be preferable due to its increased axial strain and limited lateral displacements. Similar preliminary investigations have also been conducted for acoustic radiation force based shear wave elasticity imaging^23^, in which motion correction is not feasbile due to instant generation of the shear wave.

The hypothesis of this study is that the longitudinal view may be more reliable for imaging microvascular blood flow in thyroid since it will incur less out-of-plane motion due to the distal presence of trachea. We test this hypothesis by conducting an *in vivo* pilot study on 24 thyroid patients, across 48 imaging cross-sections. Thyroid motion is estimated using normalized cross-correlation (NCC) based 2D speckle tracking, which has been the gold standard for ultrasound based motion tracking^24^ in blood flow imaging^25^, elastographic imaging^26–28^, temperature imaging^29^, phase-aberration correction^30^. Estimated axial and lateral displacement are subsequently used for registration of the Doppler frames under the assumption of rigid body motion^14^. Presence of elevational motion is detected based on the estimates of normalized cross-correlation co-efficient of the Doppler ensemble^31–33^.

## Results

Representative images of *in vivo* thyroid sonograms across longitudinal and transverse views are displayed in Fig. 2(a,b), respectively. The outline of the thyroid nodule in longitudinal **(a)** and transverse **(b)** views, are shown in white. The corresponding PD images visualize the blood flow signal in the intra- and peri-nodular regions for longitudinal **(c)** and transverse **(d)** cross-sections, without **(c, e)** and with **(d, f)** motion correction. For the PD image obtained from the longitudinal cross-section, the flow signal was visibly corrupted by a horizontal motion blur **(c)**, which upon motion correction using the approach outlined in Fig. 1(d), displayed a noticeable improvement in the visualization of the blood vessels **(e)**. However in the transverse view, applying motion correction to the Doppler ensemble **(d)** produced no visible improvement **(f)**. Particularly in the transverse view, as expected from the anatomical orientation illustrated in Fig. 1(b), the location of the trachea (right), thyroid (center) and the carotid artery (left) was visible. Correspondingly in the PD images, the carotid artery in the transverse view can be observed to display a large cross-section of flow, adjacent to another blood vessel. However, in the longitudinal view, the trachea and the carotid artery were not visible, due to their distal and proximal location with respect to the thyroid, respectively.

**Figure 3:**
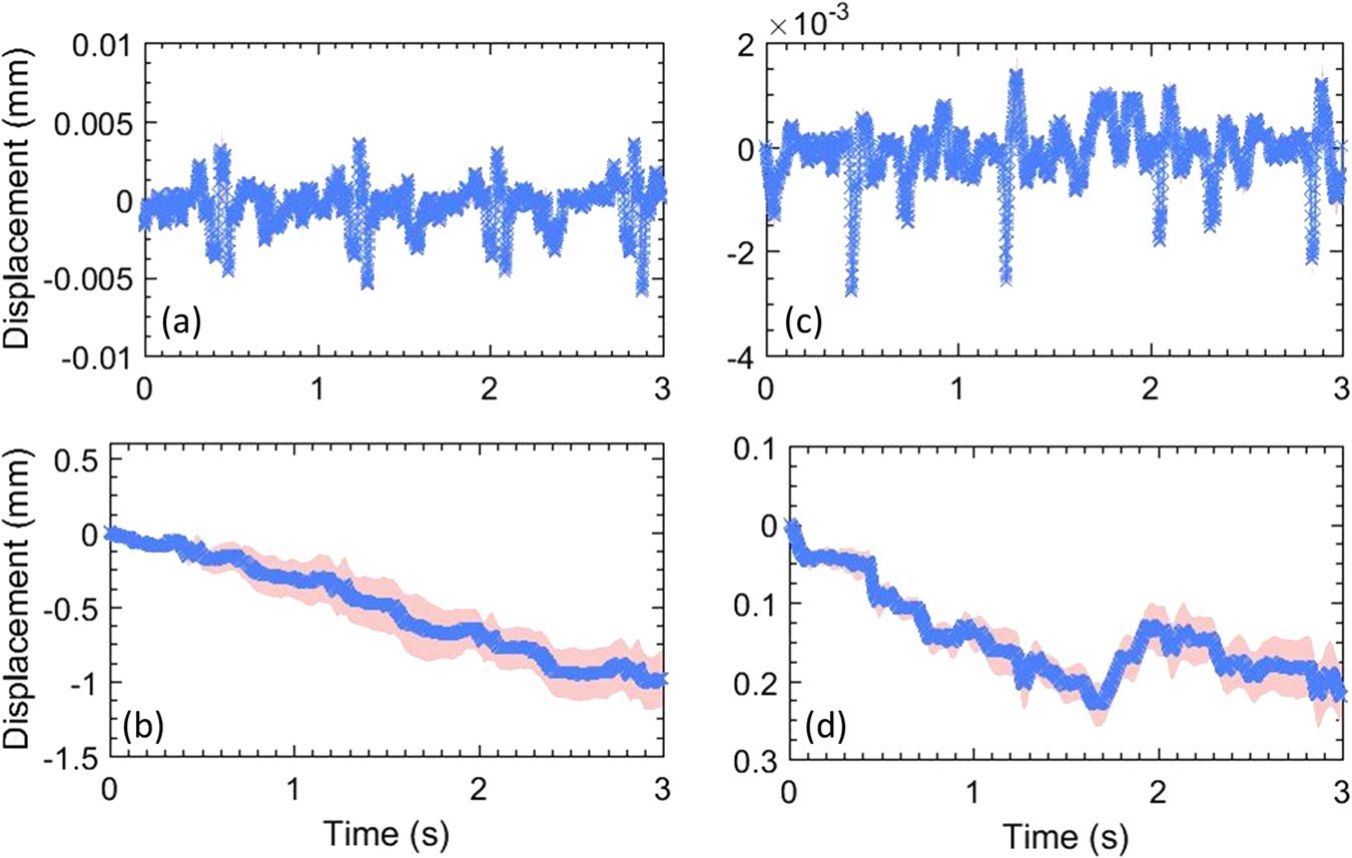
(**a–d**) display the lateral and axial displacements, respectively, estimated in the longitudinal view of the thyroid. Specifically, (**a**,**c**) show the displacements estimated between consecutive frames, and (**b**,**d**) show the total accumulated displacements, with reference to the first frame in the ensemble. The continuous error-band (red) displays ±1 standard deviation from the mean.

**Figure 4:**
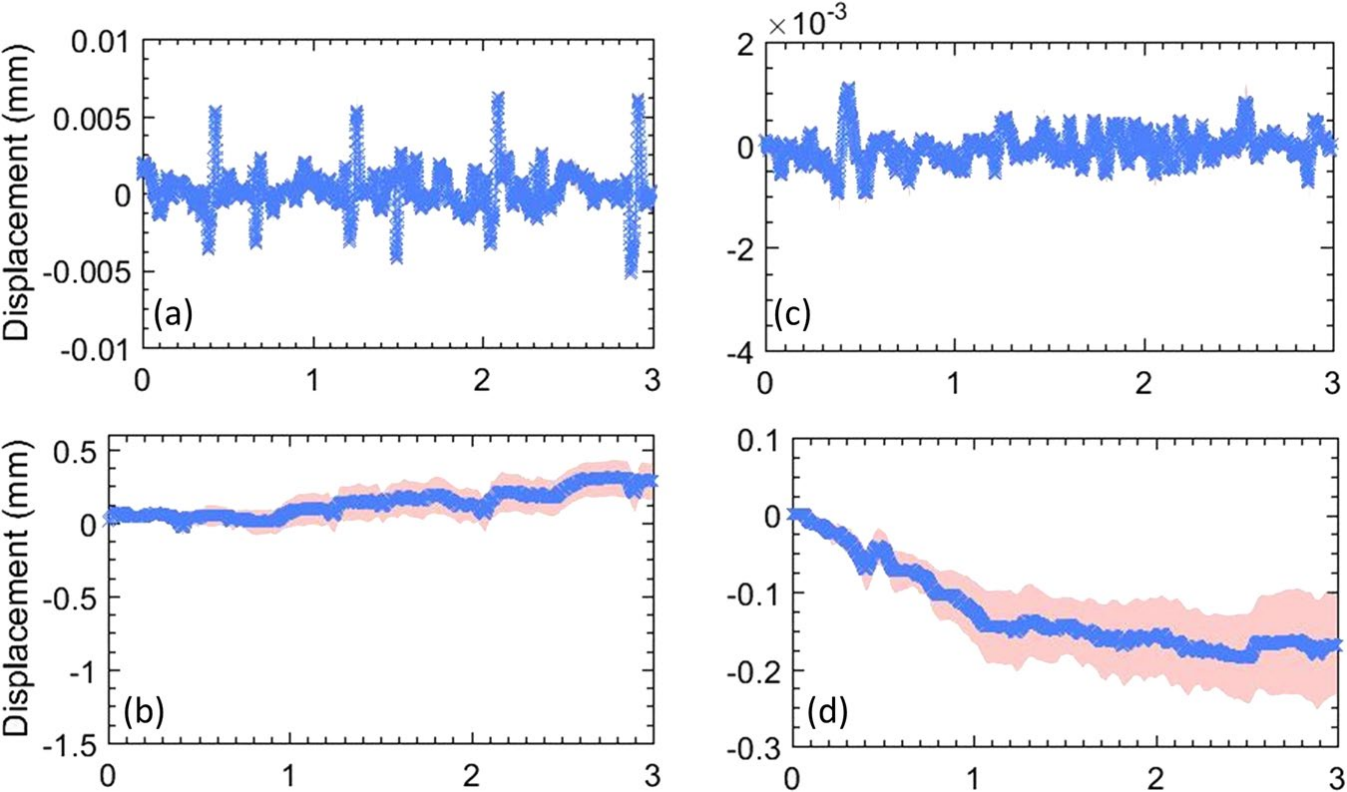
(**a**–**d**) display the lateral and axial displacements, respectively, estimated in the transverse view of the thyroid. Specifically, (**a**,**c**) show the displacements estimated between consecutive frames, and (**b**,**d**) show the total accumulated displacements, with reference to the first frame in the ensemble. The continuous error-band (red) displays ±1 standard deviation from the mean.

Figures 3 and 4 display the motion estimated in thyroid across longitudinal and transverse views, respectively. The inter-frame tissue motion estimated between consecutive frames were computed as components of lateral **(a)** and axial **(b)** displacements, using 2D cross-correlation based speckle tracking. The normalized cross-correlation co-efficient averaged over the thyroid tissue between consecutive frames were consistently 0.97. **(a,c)** display the mean lateral and axial displacements associated with the entire thyroid nodule, respectively, as a function of time. To estimate the mean displacements (blue), the thyroid motion was assumed to be rigid and translational. The estimated standard deviation (red) in **(a,c)** was negligible, since the variation of axial and lateral displacements in the nodule were small. This also indicates that the lesion experienced low strain, which otherwise would lead to a strong displacement gradient, and increased variance. The estimated axial and lateral displacements were integrated in slow-time to compute the net instantaneous displacement of the thyroid lesion **(b,d)**, which was subsequently used for motion correction. The estimated standard deviation was observed to increase upon displacement accumulation **(b,d)** due to increase in number of frames. Further, the signature of carotid pulsation was visible in the lateral displacement estimates **(a)**, which was expected given the anatomical orientation of the thyroid relative to carotid artery. These observations were similar to our previous report on feasibility of motion correction in thyroid nodules^14,34^. Lateral motion of the thyroid lesion was noticeably higher in the longitudinal view, compared to transverse. Specifically, in the transverse view magnitude of thyroid motion in the axial and lateral directions were comparable, whereas, in the longitudinal view, lateral motion predominated. Further, the estimated displacements **(b,d)** also displayed presence of translational motion, which could be due to the slipping of the nodule or the probe during scanning^14,34^.

**Figure 5:**
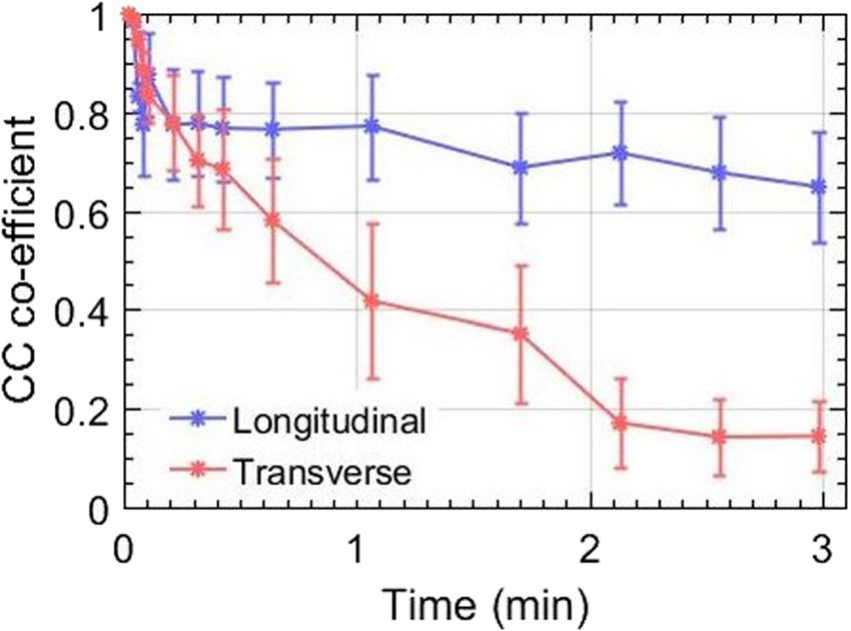
Displays the average correlation co-efficient of the Doppler frames with respect to the reference frame in longitudinal and transverse views. The corresponding error-bars display ±1 standard deviation from the mean. The cross-correlation coefficients were normalized between 0 and 1.

Figure 5 displays the mean cross-correlation coefficient of the entire Doppler ensemble with respect the to the reference frame (i.e the first frame in the ensemble), across both longitudinal (blue) and transverse (red) views. The ensemble cross-correlation co-efficients, normalized between 0 and 1, are an important indicator of the frame similarity across the entire ensemble. Frames associated with in-plane translational motion display high ensemble cross-correlation coefficient, approaching unity. Low ensemble cross-correlation coefficients can be indicative out-of-plane motion or speckle decorrelation due to high strain. In both, longitudinal and transverse planes, a steady decay in the ensemble cross-correlation was visible which was expected, since perfect in-plane motion may not be feasible in *in vivo* conditions. However, the decay in cross-correlation coefficient in the transverse plane was considerably large (0.2985 ± 0.0724) compared to that in the longitudinal plane (0.6488 ± 0.1121). This was possibly due to the presence of the rigid trachea, which restricted lateral motion but reciprocally increased elevational motion.The error bars corresponds to ±1 standard deviation.

**Figure 6:**
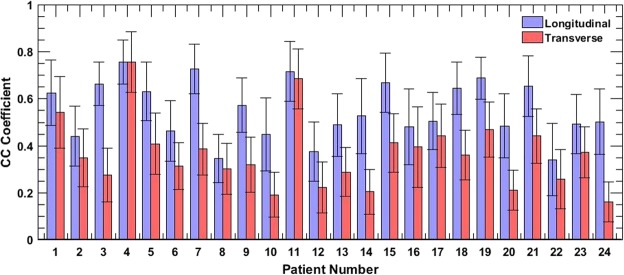
Displays the average ensemble cross-correlation associated with thyroid lesions of 24 patients, in longitudinal and transverse views. The errorbars correspond to ±1 standard deviation.

**Figure 7:**
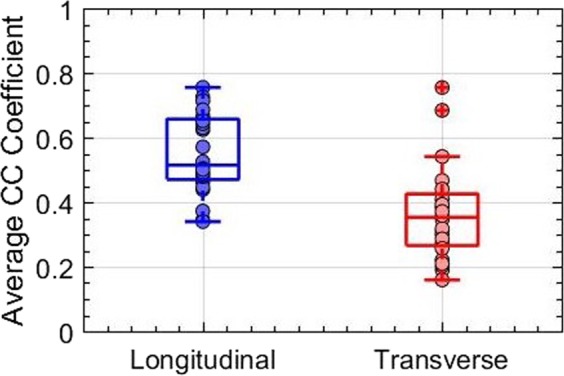
Displays boxplot of the average ensemble cross-correlation associated with thyroid nodules of 24 patients, in the longitudinal and transverse views.

The bar plot in Fig. 6 displays the normalized ensemble cross-correlation coefficients, averaged over the thyroid nodule, for the entire study population of 24 patients, across 48 acquisitions. The ensemble cross-correlation coefficients were considerably higher in the longitudinal view than in the corresponding transverse view. While Fig. 6 directly compares the cross-correlation coefficient associated with the two views, Fig. 7 displays the pooled distribution for the entire population, with the 48 acquisitions categorized into longitudinal (blue) and transverse (red) views. The median ensemble cross-correlation for the entire patient population in the longitudinal and transverse planes were 0.51 and 0.352, respectively. Further, the maximum and minimum cross-correlation coefficients were (0.755, 0.339) in the longitudinal view, and (0.754, 0.159) in the transverse. Although the maximum values were similar for the two cross-sections, however, 25^*th*^ and 75^*th*^ percentile values were (0.470, 0.51) in the longitudinal and (0.352, 0.425) in the transverse views. The pooled data displayed that the ensemble cross-correlation coefficients in the longitudinal view was significantly higher (p < 0.05) than in the transverse, suggesting that the thyroid nodule in the transverse cross-section incurred increased elevational motion than in the longitudinal view.

**Figure 8:**
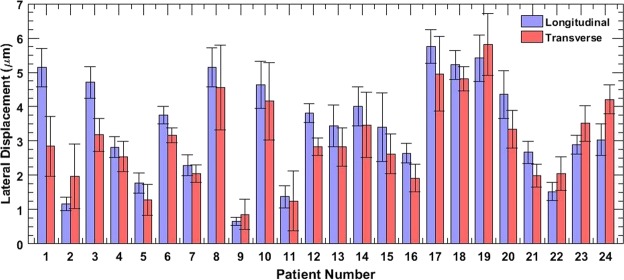
Displays the average lateral displacements associated with thyroid lesion of 24 patients, in longitudinal and transverse views. The errorbars correspond to ±1 standard deviation.

The barplots in Fig. 8 display the mean lateral displacements associated with the thyroid nodule in the longitudinal (blue) and transverse (red) cross-sections, for 24 patients, across 48 cross-sections. The mean displacements corresponds to the entire thyroid nodule, and the errorbars show ±1 standard deviation. The result show that in 75% of the cases, the mean lateral displacements of the thyroid nodule were larger in the longitudinal view than in the transverse. The absolute displacements across patients varied due to multiple factors such as amplitude of the carotid pulsations, size and stiffness of the lesion and the location and distance of the thyroid nodule from the carotid^14^.

**Figure 9:**
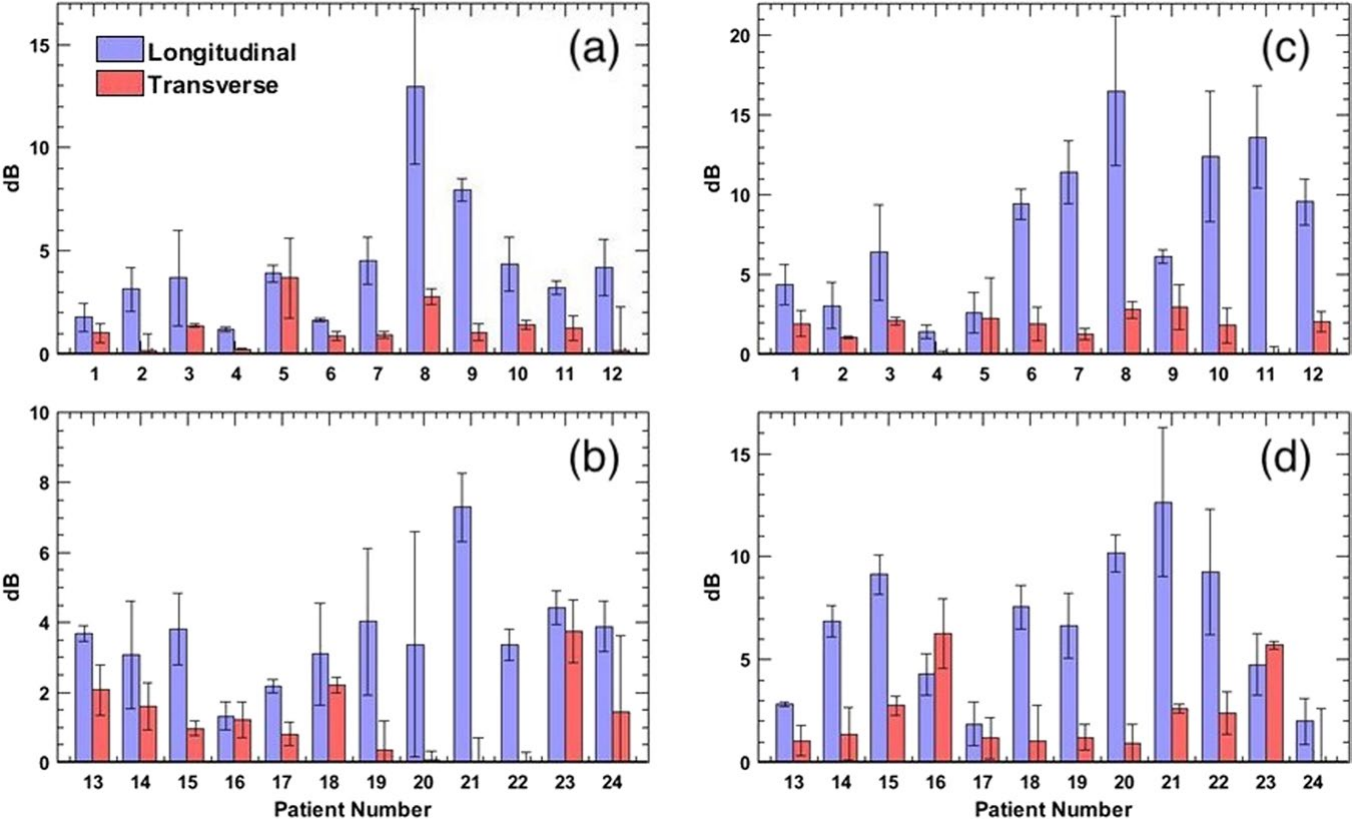
Displays barplots of increase in CNR and SNR of the PD images of 24 patients upon motion correction of the Doppler ensemble from longitudinal and transverse views. The left (**a**,**b**) and right (**c**,**d**) column corresponds to CNR and SNR barplots, respectively.

The barplots in Fig. 9 display improvement CNR and SNR of the PD images, in longitudinal and transverse views, upon motion correction. These quantitative metrics were estimated using Eqs. 3 and 4, as described in^14,34^, with and without the correction of thyroid motion. The reported error-bars were computed from three blood vessel ROIs, which intrinsically varied in flow intensity, size and depth^14,34^. The absolute SNR and CNR varied across the patient population and the imaging views, as presented in supplementary data. Figure 9 displays that the increase in the CNR and SNR upon motion correction was considerably higher in the longitudinal view than in transverse. Further, the increase in CNR and SNR upon motion correction were 12.95 ± 3.76 dB and 16.48 ± 4.6 dB, respectively, in the longitudinal cross-section. Correspondingly in the transverse cross-section, increase in CNR and SNR upon motion correction were 3.72 ± 0.894 dB and 6.217 ± 1.689 dB, respectively.

## Methods

### Data acquisition

The *in vivo* ultrasound imaging of the thyroid was performed using an Alpinion E-Cube 12R scanner (Alpinion Medical Systems Co., Seoul, South Korea). Ultrasound in-phase and quadrature (IQ) data was acquired with a linear array probe (L12-3H) that transmitted and received using 128 and 64 elements, respectively. Compounded plane wave imaging was performed with 7 insonification angles, with maximum of ±3° ^35–37^. Ultrasound imaging was performed at a transmit frequency of 11.5 MHz, and sampling rate of 40 MHz. The data acquisition window was three seconds, and the frame-rate varied with depth of imaging, depending on location and size of the nodule. In the beam-formed image, each pixels were of size 38.5 *μ*m and 200 *μ*m in the axial and lateral directions, respectively. The speed of sound was assumed to be 1540 m/s for the calculation of transmission and beam-forming delays.

### *In vivo* study

The *in vivo* study was conducted to evaluate influence of imaging cross-section on the visualization of blood flow signal in the thyroid. The pilot study was conducted on 24 patients, with at least one suspicious thyroid nodule recommended for US-guided FNA biopsy. This study was approved by the Mayo Clinic institutional review board, and the methods were carried out in accordance with the relevant guidelines and regulations. An approved informed consent in written was obtained from all patients prior to their participation. Although, a majority of the recruited subjects were females, we don’t expect any impact of gender on the outcome of this study. The ultrasound data was acquired by an experienced sonographer, across the longitudinal and transverse cross-sections. All ultrasound scans were performed an hour before the scheduled FNA biopsy. To minimize the impact of breathing on the acquired data, the subject was asked to hold her breath for the 3 seconds duration of the image acquisition. For all 24 patients, the frame-rate was same across longitudinal and transverse plane, since the imaging depth and location of the nodule was the same. Though the frame-rate varied across patients, relative to the size and location of the thyroid nodule; we do not expect that to have any impact on the results of this study, since the results are specifically compared across the two imaging cross-sections for each patient.

### Displacement tracking

Motion associated with the thyroid nodule was tracked using 2D normalized cross-correlation based speckle tracking. The axial and lateral displacement were estimated prior to clutter-filtering, and were subsequently used for motion correction of clutter-filtered ensembles. The size of the 2D kernel used for displacement estimation was 0.2 mm × 0.8 mm, which overlapped by 90% in both coordinates. Sub-pixel displacements were estimated using a 2D spline interpolator. Further, the IQ ultrasound images were interpolated by a factor of 3 and 10, in the axial and lateral directions, respectively, to increase the spatial density of correlation functions^38–40^. The 2D cross-correlation based displacement tracking was performed on a Titan XP GPU (Nvidia Corp., CA, US). Displacements were tracked between every consecutive frame, and the estimated axial and lateral displacements maps were transformed from Eulerian to Lagrangian co-ordinates, to correspond with the first frame of the Doppler ensemble. For estimation of ensemble cross-correlation, the above defined 2D kernel was used for cross-correlating the first-frame with every other frame in the ensemble.

### SVD based spatio-temporal clutter filtering

The ultrasound data was clutter filtered using the singular value decomposition of the spatiotemporal Casaroti matrix^12^.

1 $${S}_{blood}=S(x,z,t)-\mathop{\sum }\limits_{r=1}^{r=th}{U}_{r}{\lambda }_{r}{V}_{r}^{\ast }$$

where the matrices *S* and *S*_*biood*_ represent pre- and post-clutter filtered Doppler ensemble. The matrices *U*, *V* consist of left and right singular orthonormal vectors, respectively. The corresponding singular values and their order are denoted by *λ*_*r*_ and *r*, respectively, and * represents conjugate transpose. A global SV threshold was chosen to separate tissue clutter from blood signal, based on the decay of the double derivative of the SV orders (i.e. when the double derivative approached zero).

### Motion correction

Motion correction of the Doppler ensemble was performed subsequent to clutter-suppression. This allowed registration of each ultrasound frame with that of the first frame (i.e reference frame), by globally translating the rows and columns by the estimated displacements using a spile based interpolation technique. The mean axial and lateral displacements estimated from each frame, averaged over the demarcated tumor nodule, were used to correct first-order rigid-body motion^14^. Since we expected that transverse plane may experience increased out of plane motion, performing motion correction prior to clutter filtering could have hampered reliable tissue suppression in the transverse plane compared to longitudinal plane^34^. Therefore, motion correction prior to clutter-filtering was avoided to prevent the negative impact of erroneous motion tracking and correction on the performance of clutter filtering.

### Power doppler imaging

The final power Doppler signal was computed from the motion corrected data:

2 $$PD(x,z)=\mathop{\sum }\limits_{t=1}^{{N}_{t}}{|{S}_{blood}(x,z,t)|}^{2}$$

where *N*_*t*_ denotes the ensemble length. The background separation in the PD image was achieved by using a top-hat morphological filter, with a disc-shaped structuring element of radius 10 pixels^41^. Prior to top-hat filtering, the PD image image was interpolated to the equal number of rows and column to equally apply the disc-shapped structuring element along both axial and lateral direction.

### Data analysis

Quantitative assessment of the imaging performance was performed by estimating the contrast to noise ratio (CNR) and the signal to noise ratio (SNR) of the PD images:

3 $$CNR=20{lo}{{g}}_{10}(\frac{|{\mu }_{v}-{\mu }_{bg}|}{\sqrt{{\sigma }_{v}^{2}+{\sigma }_{bg}^{2}}})$$

4 $$SNR=20{lo}{{g}}_{10}(\frac{{\mu }_{v}}{{\mu }_{bg}})$$

where *μ* and *σ* denotes the mean and the standard deviation of the signal, respectively. Further, the subscripts *v* and *bg* corresponds to signal obtained from the vessel and background regions, respectively. A constant offset of 10 dB was added to all estimated CNR values to display a positive estimate, specifically, in the absence of motion correction.

## Conclusion

The motion from the carotid artery can noticeably impact power Doppler imaging of small vessels in thyroid. The results obtained in this study demonstrate that thyroid motion could be reliably tracked and corrected in the longitudinal view than in the transverse, which is important for accurate visualization of blood flow. This pilot study was conducted using a commercial ultrasound scanner implemented with compounded plane wave imaging, on 24 human subjects with thyroid nodules suspicious of malignancy. This study is particularly valuable in contrast-free power Doppler imaging, since tissue motion can easily corrupt the low intensity of the flow signal. The preliminary results obtained from this pilot study will be useful in further *in vivo* validation in a larger patient population.

## Supplementary information

Supplementary Information.

## References

[1] Moon W-J (2008). Benign and malignant thyroid nodules: Us differentiation—multicenter retrospective study. Radiology.

[2] Appetecchia M, Solivetti F (2006). The association of colour flow doppler sonography and conventional ultrasonography improves the diagnosis of thyroid carcinoma. Hormone Res. Paediatrics.

[3] Bae U (2007). Ultrasound thyroid elastography using carotid artery pulsation. J. Ultrasound Med..

[4] Brunese L (2008). A new marker for diagnosis of thyroid papillary cancer. J. Ultrasound Med..

[5] Cerbone G (1999). Power doppler improves the diagnostic accuracy of color doppler ultrasonography in cold thyroid nodules: follow-up results. Hormone Res. Paediatrics.

[6] Chan BK, Desser TS, McDougall IR, Weigel RJ, Jeffrey RB (2003). Common and uncommon sonographic features of papillary thyroid carcinoma. J. Ultrasound Med..

[7] Frates MC, Benson CB, Doubilet PM, Cibas ES, Marqusee E (2003). Can color doppler sonography aid in the prediction of malignancy of thyroid nodules?. J. ultrasound Med..

[8] Lu R (2017). Superb microvascular imaging (smi) compared with conventional ultrasound for evaluating thyroid nodules. BMC Med. imaging.

[9] Lyshchik A (2005). Thyroid gland tumor diagnosis at us elastography. Radiology.

[10] Papini E (2002). Risk of malignancy in nonpalpable thyroid nodules: predictive value of ultrasound and color-doppler features. J. Clin. Endocrinol. & Metab..

[11] Varverakis E, Neonakis E, Tzardi M, Chrysos E (2007). Role of color doppler ultrasonography in preoperative management of cold thyroid nodules. HORMONES-ATHENS.

[12] Demené C (2015). Spatiotemporal clutter filtering of ultrafast ultrasound data highly increases doppler and fultrasound sensitivity. IEEE Trans. Med. imaging.

[13] Baranger, J. *et al*. Adaptive spatiotemporal svd clutter filtering for ultrafast doppler imaging using similarity of spatial singular vectors. *IEEE Transactions on Medical Imaging* (2018). 10.1109/TMI.2018.2789499 29969408

[14] Nayak R (2018). Non-contrast agent based small vessel imaging of human thyroid using motion corrected power doppler imaging. Sci. Rep..

[15] Dighe M (2008). Differential diagnosis of thyroid nodules with us elastography using carotid artery pulsation. Radiology.

[16] Bhatia K (2011). Cystic change in thyroid nodules: a confounding factor for real-time qualitative thyroid ultrasound elastography. Clin. radiology.

[17] Ning C-P (2012). The value of strain ratio in differential diagnosis of thyroid solid nodules. Eur. J. radiology.

[18] Oliver, C. *et al*. What is the contribution of elastography to thyroid nodules evaluation? In *Annales d’endocrinologie*, vol. 72, 120–124 (Elsevier, 2011). 10.1016/j.ando.2011.03.016 21513909

[19] Park M (2009). Sonography of thyroid nodules with peripheral calcifications. J. Clin. Ultrasound.

[20] Park SH (2009). Interobserver agreement in assessing the sonographic and elastographic features of malignant thyroid nodules. Am. J. Roentgenology.

[21] Huang, Y. *et al*. Diagnostic performance of ultrasound strain elastography in transverse and longitudinal views in predicting malignant thyroid nodules. *Ultrasound in Medicine & Biology* (2019). 10.1016/j.ultrasmedbio.2019.05.018 31196745

[22] Cosgrove D (2017). Wfumb guidelines and recommendations on the clinical use of ultrasound elastography: Part 4. thyroid. Ultrasound Med. & Biol..

[23] Bhatia KS (2012). Shear wave elastography of thyroid nodules in routine clinical practice: preliminary observations and utility for detecting malignancy. Eur. radiology.

[24] Viola F, Walker WF (2003). A comparison of the performance of time-delay estimators in medical ultrasound. IEEE Trans. ultrasonics, ferroelectrics, frequency Control..

[25] Trahey, G. E., Allison, J. W. & Von Ramm, O. T. Angle independent ultrasonic detection of blood flow. *IEEE Transactions on Biomedical Engineering* 965–967 (1987). 10.1109/tbme.1987.325938 2961682

[26] Ophir J (2002). Elastography: imaging the elastic properties of soft tissues with ultrasound. J. Med. Ultrason..

[27] Greenleaf JF, Fatemi M, Insana M (2003). Selected methods for imaging elastic properties of biological tissues. Annu. Rev. Biomed. Eng..

[28] Parker KJ, Doyley MM, Rubens DJ (2010). Imaging the elastic properties of tissue: the 20 year perspective. Phys. Med. &amp; Biol..

[29] Varghese T, Zagzebski J, Lee F (2002). Elastographic imaging of thermal lesions in the liver *in vivo* following radiofrequency ablation: preliminary results. Ultrasound Med. Biol..

[30] Ng GC, Worrell SS, Freiburger PD, Trahey GE (1994). A comparative evaluation of several algorithms for phase aberration correction. IEEE Trans. ultrasonics, ferroelectrics, frequency Control..

[31] Walker WF, Trahey GE (1995). A fundamental limit on delay estimation using partially correlated speckle signals. IEEE Trans. Ultrasonics, Ferroelectrics, Frequency Control..

[32] Varghese T, Ophir J (1997). A theoretical framework for performance characterization of elastography: The strain filter. IEEE Trans. ultrasonics, ferroelectrics, frequency Control..

[33] Jiang J, Hall TJ, Sommer AM (2006). A novel performance descriptor for ultrasonic strain imaging: A preliminary study. IEEE Trans. ultrasonics, ferroelectrics, frequency Control..

[34] Nayak R, Kumar V, Webb J, Fatemi M, Alizad A (2019). Non-invasive small vessel imaging of human thyroid using motion-corrected spatiotemporal clutter filtering. Ultrasound Med. & Biol..

[35] Montaldo G, Tanter M, Bercoff J, Benech N, Fink M (2009). Coherent plane-wave compounding for very high frame rate ultrasonography and transient elastography. IEEE Trans. Ultrasonics, Ferroelectr. Frequency Control..

[36] Nayak R, Schifitto G, Doyley MM (2017). Noninvasive carotid artery elastography using multielement synthetic aperture imaging: Phantom and *in vivo* evaluation. Med. Phys..

[37] Denarie B (2013). Coherent plane wave compounding for very high frame rate ultrasonography of rapidly moving targets. IEEE Trans. Med. Imaging.

[38] Parker JA, Kenyon RV, Troxel DE (1983). Comparison of interpolating methods for image resampling. IEEE Trans. Med. imaging.

[39] Konofagou E, Ophir J (1998). A new elastographic method for estimation and imaging of lateral displacements, lateral strains, corrected axial strains and poisson’s ratios in tissues. Ultrasound Med. Biol..

[40] Huntzicker S, Nayak R, Doyley MM (2014). Quantitative sparse array vascular elastography: The impact of tissue attenuation and modulus contrast on performance. J. Of. Med. Imaging.

[41] Bayat M, Fatemi M, Alizad A (2018). Background removal and vessel filtering of noncontrast ultrasound images of microvasculature. IEEE Trans. Biomed. Eng..

