# Author Correction: Impact of imaging cross-section on visualization of thyroid microvessels using ultrasound: Pilot study

**DOI:** 10.1038/s41598-020-69042-7

**Published:** 2020-07-15

**Authors:** Rohit Nayak, Noshin Nawar, Jeremy Webb, Mostafa Fatemi, Azra Alizad

**Affiliations:** 1 Department of Radiology, Mayo Clinic College of Medicine and Science, Rochester, MN 55905 USA; 2 Department of Physiology and Biomedical Engineering, Mayo Clinic College of Medicine and Science, Rochester, MN 55905 USA

Correction to: *Scientific Reports* https://doi.org/10.1038/s41598-019-57330-w, published online 15 January 2020.

This Article contains an error where the Discussion section has been inadvertently omitted. As a result of this, 19 references were also omitted. The correct Discussion section appears below.

## Discussion

Carotid pulsations can significantly degrade the visualization of vascular pathway in the thyroid, especially of small blood vessels. In a recent publication^1^, we demonstrated that PD images corrupted by motion could be noticeably improved by motion correction. However, this is feasible only if the thyroid motion in-plane that can be reliably tracked using ultrasound speckle tracking techniques. Anatomically, thyroids glands are intrinsically bound by a rigid trachea and a pulsating carotid artery at opposite ends, which influences its motion characteristics and its scope for correction across different imaging planes. The goal of this pilot study is to investigated the efficacy of motion correction for thyroid microvascular imaging across the transverse and longitudinal cross-sections.

The preliminary results obtained on 24 patients, involving 48 image acquisitions demonstrate that tracking of thyroid motion in the longitudinal view was more reliable than than in the transverse. In-plane thyroid motion was primarily visible in the longitudinal view [Figs. 2, 5 -7], owing to the distal presence of rigid trachea that restricted elevational motion. Further, thyroid motion in the longitudinal plane was associated with a strong lateral component of displacement [Figs. 2, 3, 8]. Reciprocally in the transverse view, presence of trachea limited lateral motion [Figs. 2, 4 and 8] but increased out-of-plane incursions [Figs. 5–7]^2^. However, in-plane motion can be reliably tracked in terms of axial and lateral components, and subsequently corrected. Whereas, elevational motion leads to out-of-plane excursions that cannot be accounted using 2D ultrasound. This was also observed in the CNR and SNR barplots in Fig. 9, the improvements in signal quality was higher in the longitudinal direction compared to transverse. Although absolute CNR and SNR varied across patients and cross-sections depending on flow intensity of the chosen vessel, attenuation at the imaging depth, and size of the acquired Doppler ensemble, however, improvement in PD image quality was more prominent in the acquisitions obtained from the longitudinal view. This is consistent with the observation that ensemble correlation coefficient was also higher in the longitudinal plane, suggesting motion was reliably tracked for subsequent correction. Ideally, in the absence of motion, we don’t expect one imaging cross-section to display higher microvascular signal quality than other. However, in this paper, comparison of absolute CNR and SNR of the same vessel across the two imaging planes was not possible without real-time 2D imaging capabilities for vessel registration or 3D volumetric imaging. Therefore direct comparison of absolute estimates of CNR and SNR across longitudinal and transverse cross-section may not be appropriate.

Cross-correlation coefficient has been commonly used in strain elastography to detect speckle decorrelation, which is typically occurs due to (1) out-of-plane motion or (2) at very large strains (> 10%). The correlation coefficient associated with frame-to-frame tracking was consistently > 0.97, regardless of the imaging cross-section, which was specifically due to high frame-rate of imaging. However, results obtained from matching the reference frame with other ensemble frames displayed a steep decay in cross-correlation coefficient in the transverse view than in the longitudinal view [Fig. 5]. This observation was consistent across all the 48 acquisitions [Fig. 5], and the pooled distribution demonstrated that the normalized ensemble cross-correlation—which is indicative increased speckle decorrelation—was significantly higher (p < 0.05) in the longitudinal view than in the transverse, in the later.

In strain elastography, WFUMB guidelines recommend imaging of the thyroid in the longitudinal view^3^. This is based on the anatomical orientation of the thyroid with respect to the rigid trachea and the pulsating carotid artery, and its surrounding tissue. With recent developments in motion-robust clutter-filtering^1,4–6^, a suitable investigation to assess the impact of imaging cross-section on feasibility of thyroid microvasculature imaging can be useful in successfully translating it to the clinic for non-invasive diagnosis. An important question is: What are the disastrous consequences of imaging thyroid microvasculature with increased out-of-plane motion? Three obvious consequences are that this could (i) substantially hamper any efforts in motion tracking and correction, in the absence of 3D ultrasound imaging that is currently at a very nascent stage of development and implementation^7^; (ii) would require advanced performance metrics for real-time assessment of the coherence of the acquired data to identify frames subjected to out-of-plane motion, which must be discarded prior to slow-time PD integration^6,8^. Further, discarding frames from the Doppler ensemble will inadvertently hamper the quality of the PD image, especially since flow signal in ultrasound images are only marginally higher than the noise floor^1^; (iii) would complicate implementation of real-time imaging—a crucial feature of 2D ultrasound—for comprehensive assessment of the thyroid microvasculature in the entire nodule^9^.

High frame-rate imaging is expected to reduce the impact of motion on thyroid microvasculature imaging. However, size and location of the tumor can impact depth of imaging, which is inversely proportional to frame-rate. Further, multi-angle plane-wave insonifications required for compounded plane wave imaging also is an important factor that decides the image quality, and influences the frame-rate. Increasing the number of angular insonification can considerably reduce the impact of grating lobes, and improve the signal-to-noise ratio of the received signal^10^. Further, the angular configurations of compounding plane wave imaging is also an important factor that determines the efficacy of the motion tracking, especially in the lateral direction^10^. Additionally, the capability of ultrafast imaging is currently available in limited high-end ultrasound systems. The motion corrected PD microvasculature imaging technique can be valuable in improving the performance of mid-range clinical scanners that multiplex repeated acquisitions due to reduced number of receive channels, which can affect imaging frame-rate. Specifically, this study was conducted using an Alipinion E-Cube 12R clinical ultrasound system, which acquired received data of 128 channels with only 64 elements at a time, therefore reducing the imaging frame-rate by half. Even at high imaging frame-rate, motion correction can be valuable in acquiring and concatenating Doppler ensembles to produce an ultra-long, coherent ensemble than can substantially boost the signal strength of small vessels at depth, which may get degraded due to ultrasound attenuation.

Conventionally, lack of phase information in the direction perpendicular to ultrasound beam propagation makes estimation of lateral motion very challenging. Several studies have been conducted to advance tracking and beam-forming techniques to improve the robustness of lateral motion tracking using ultrasound^10–14^. The in-plane axial and lateral displacements estimated across the 48 cross-sections varied across subjects depending on factors such as the size and stiffness of the nodule and its bonding with the surrounding, it location in the thyroid and its proximity to the carotid artery [Fig. 8]. Further, the amplitude of the carotid pulsation that depends on pulse pressure and the stiffness of the artery, will also determine the displacements incurred by the thyroid nodule^12,15^. Further, the axial and lateral motion estimated in the thyroid nodule using the 2D normalized cross-correlation were similar to those reported in carotid elastography studies^16,17^.

A limitation of our current study is that a first-order global motion correction approach was used for correcting thyroid motion, under the assumption of rigid body translations, which may not be accurate. However, since the variance in frameto- frame displacements obtained in the target nodules [Figs. 3 (a, c) and 4 (a, c)] were observed to be low, we used such an approach for this pilot investigation. Similar motion correction techniques based on rigid-body assumption have also been used in previous studies^1,6^, including contrast-enhanced ultrasound blood flow imaging^18,19^. In our future work, we will address this limitation by performing motion correction adaptively in local overlapping windows that can be reasonably assumed to have uniform displacements.
