# Supplementary material for: Impact of imaging cross-section on visualization of thyroid microvessels using ultrasound: Pilot study
Source: Sci Rep. 2020 Jan 15;10:415. doi: 10.1038/s41598-019-57330-w (PMC6962275; doi:10.1038/s41598-019-57330-w)
Supplement: Supplementary file 1 — Supplementary Information. [file 41598_2019_57330_MOESM1_ESM.pdf]

Supplementary Data

Impact of imaging cross-section on visualization of thyroid microvessels using ultrasound: Pilot study

Rohit Nayak[1], Noshin Nawar[1], Jeremy Webb[1], Mostafa Fatemi[2] and Azra Alizad[1]

[1]Department of Radiology, [2]Department of Physiology and Biomedical Engineering,
Mayo Clinic College of Medicine and Science, Rochester, Minnesota, 55905, United States

Figures 1, 4, 7, 10, 13 and 16 display *in vivo* thyroid sonograms and PD images across longitudinal and transverse views for patients 8, 12, 19, 21, 23, and 24, respectively. (a,b) display the B-mode sonograms corresponding to longitudinal and transverse cross-sections, respectively. The corresponding PD images visualize the blood flow signal in the intra- and peri-nodular regions for longitudinal (c) and transverse (d) cross-sections, without (c, e) and with (d, f) motion correction.

Figures 2, 5, 8, 11, 14 and 17 display the motion estimated in the thyroid longitudinal cross-section for patients 8, 12, 19, 21, 23, and 24, respectively. Figures 3, 6, 9, 12, 15 and 18 display the motion estimated in the thyroid transverse cross-section for patients 8, 12, 19, 21, 23, and 24, respectively. The inter-frame tissue motion estimated between consecutive frames were computed as components of lateral (a) and axial (b) displacements, using 2D cross-correlation based speckle tracking. The normalized cross-correlation co-efficient averaged over the thyroid tissue between consecutive frames were consistently 0.97. (a, c) display the mean lateral and axial displacements associated with the entire thyroid nodule, respectively, as a function of time. To estimate the mean displacements (blue), the thyroid motion was assumed to be rigid and translational. The estimated standard deviation (red) in (a, c) was negligible, since the variation of axial and lateral displacements in the nodule were small. This also indicates that the lesion experienced low strain, which otherwise would lead to a strong displacement gradient, and increased variance. The estimated axial and lateral displacements were integrated in slow-time to compute the net instantaneous displacement of the thyroid lesion (b, d), which was subsequently used for motion correction. The estimated standard deviation was observed to increase upon displacement accumulation (b, d) due to increase in number of frames.

Figures 19 and 20 display the absolute CNR and SNR associated with the PD images of all 24 patients, with and without motion correction in longitudinal and transverse cross-sections.

Supplementary Data

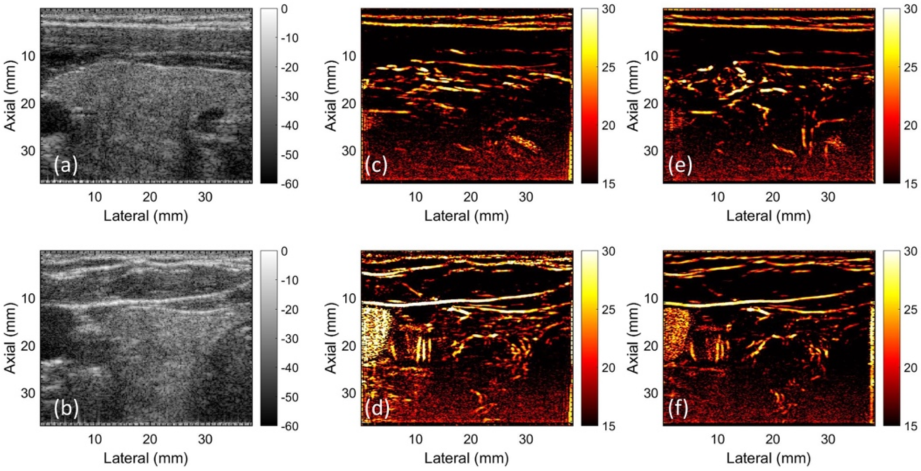

Fig. 1 displays the plane wave B-mode sonograms (a,b) and the corresponding PD images – without (c,d) and with (e,f) motion correction. (a,c,e) corresponds to the longitudinal plane, and (b,d,f) corresponds to transverse plane, for patient # 8.

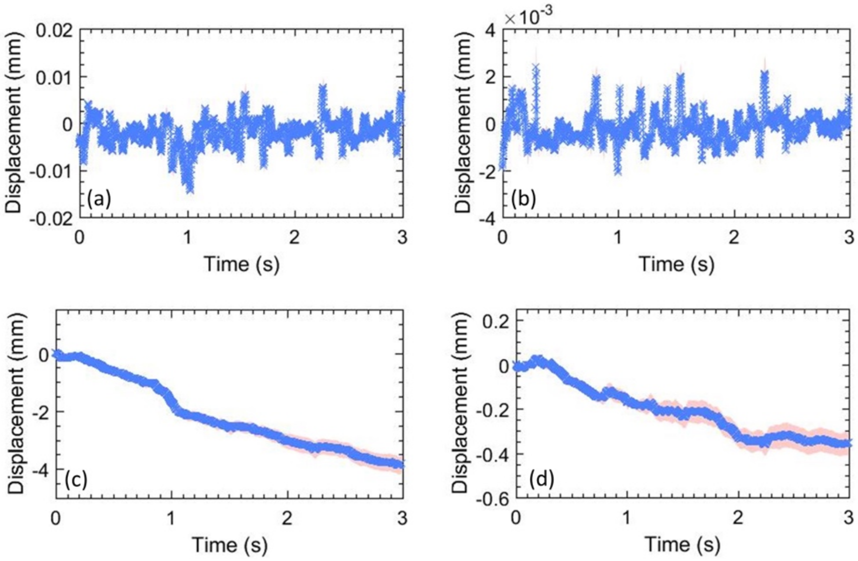

Fig. 2 (a,b) and (c,d) display the lateral and axial displacements, respectively, estimated in the longitudinal view of the thyroid for patient # 8. Specifically, (a,c) show the displacements estimated between consecutive frames, and (b,d) show the total accumulated displacements, with reference to the first frame in the ensemble. The continuous error-band (red) displays 1 standard deviation from the mean.

Supplementary Data

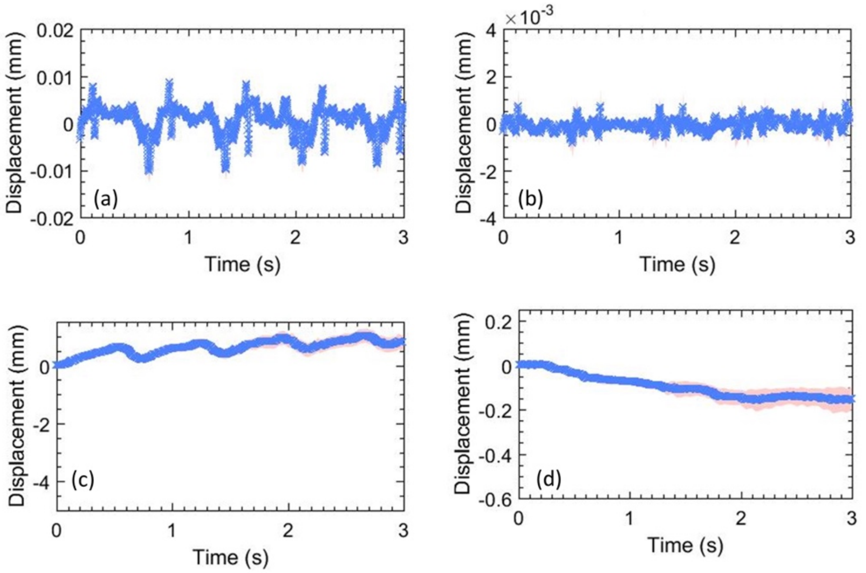

Fig. 3 (a,b) and (c,d) display the lateral and axial displacements, respectively, estimated in the transverse view of the thyroid for patient # 8. Specifically, (a,c) show the displacements estimated between consecutive frames, and (b,d) show the total accumulated displacements, with reference to the first frame in the ensemble. The continuous error-band (red) displays 1 standard deviation from the mean.

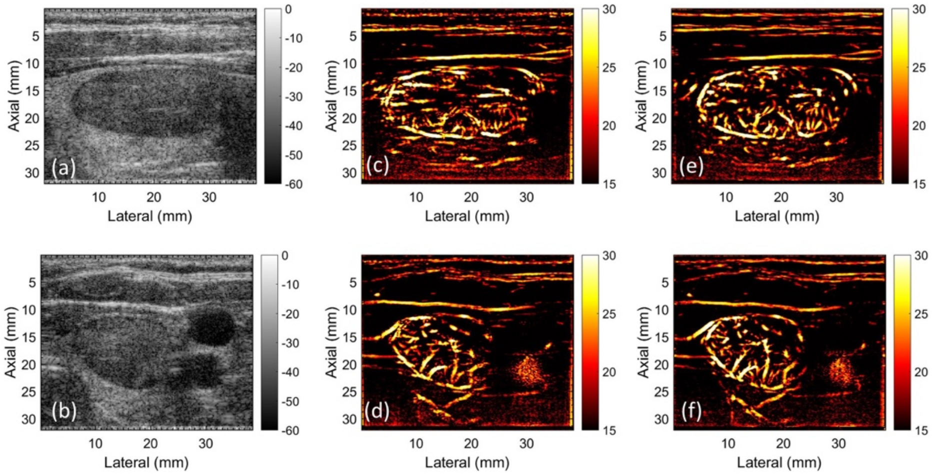

Fig. 4 displays the plane wave B-mode sonograms (a,b) and the corresponding PD images – without (c,d) and with (e,f) motion correction. (a,c,e) corresponds to the longitudinal plane, and (b,d,f) corresponds to transverse plane, for patient # 12.

Supplementary Data

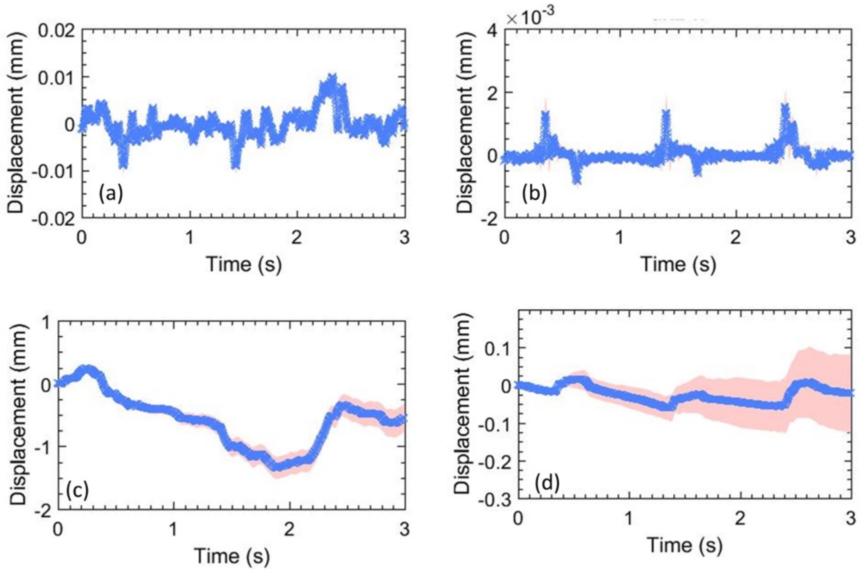

Fig. 5 (a,b) and (c,d) display the lateral and axial displacements, respectively, estimated in the longitudinal view of the thyroid for patient # 12. Specifically, (a,c) show the displacements estimated between consecutive frames, and (b,d) show the total accumulated displacements, with reference to the first frame in the ensemble. The continuous error-band (red) displays 1 standard deviation from the mean.

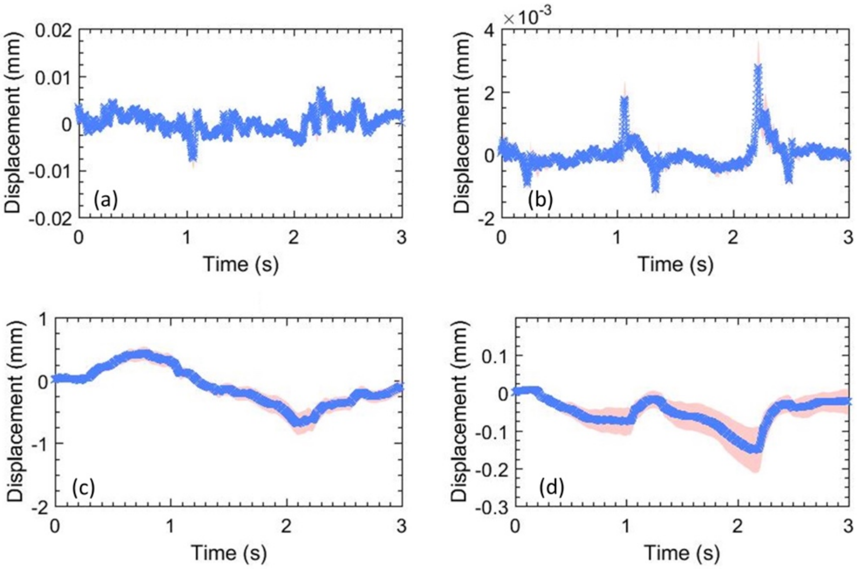

Fig. 6 (a,b) and (c,d) display the lateral and axial displacements, respectively, estimated in the transverse view of the thyroid for patient # 12. Specifically, (a,c) show the displacements estimated between consecutive frames, and (b,d) show the total accumulated displacements, with reference to the first frame in the ensemble. The continuous error-band (red) displays 1 standard deviation from the mean.

Supplementary Data

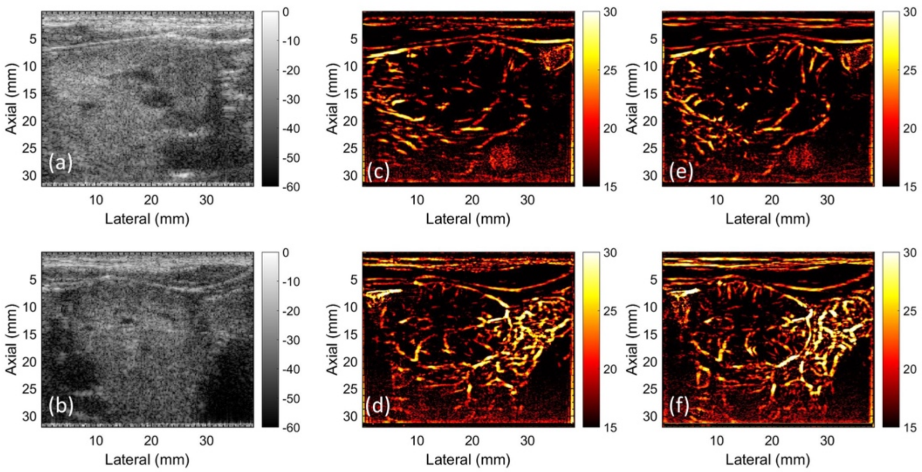

Fig. 7 displays the plane wave B-mode sonograms (a,b) and the corresponding PD images – without (c,d) and with (e,f) motion correction. (a,c,e) corresponds to the longitudinal plane, and (b,d,f) corresponds to transverse plane, for patient # 19.

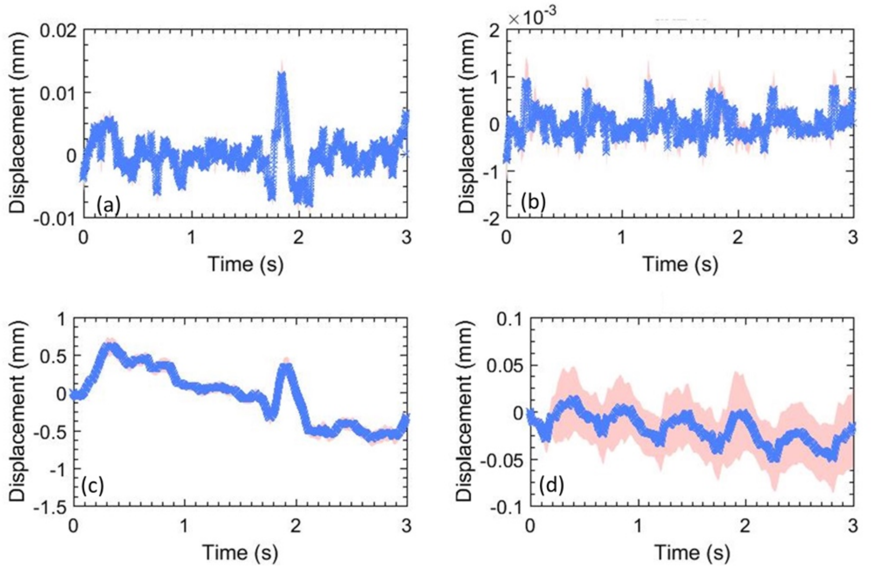

Fig. 8 (a,b) and (c,d) display the lateral and axial displacements, respectively, estimated in the longitudinal view of the thyroid for patient # 19. Specifically, (a,c) show the displacements estimated between consecutive frames, and (b,d) show the total accumulated displacements, with reference to the first frame in the ensemble. The continuous error-band (red) displays 1 standard deviation from the mean.

Supplementary Data

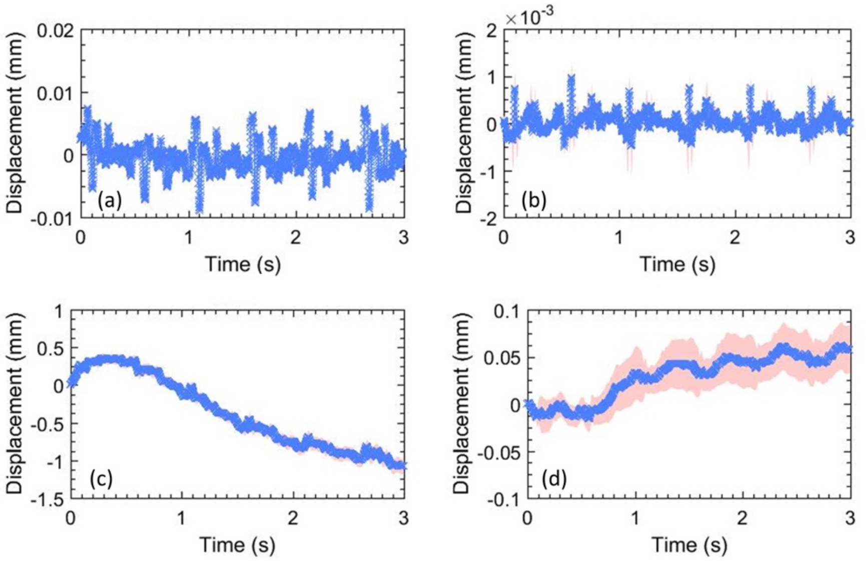

Fig. 9 (a,b) and (c,d) display the lateral and axial displacements, respectively, estimated in the transverse view of the thyroid for patient # 19. Specifically, (a,c) show the displacements estimated between consecutive frames, and (b,d) show the total accumulated displacements, with reference to the first frame in the ensemble. The continuous error-band (red) displays 1 standard deviation from the mean.

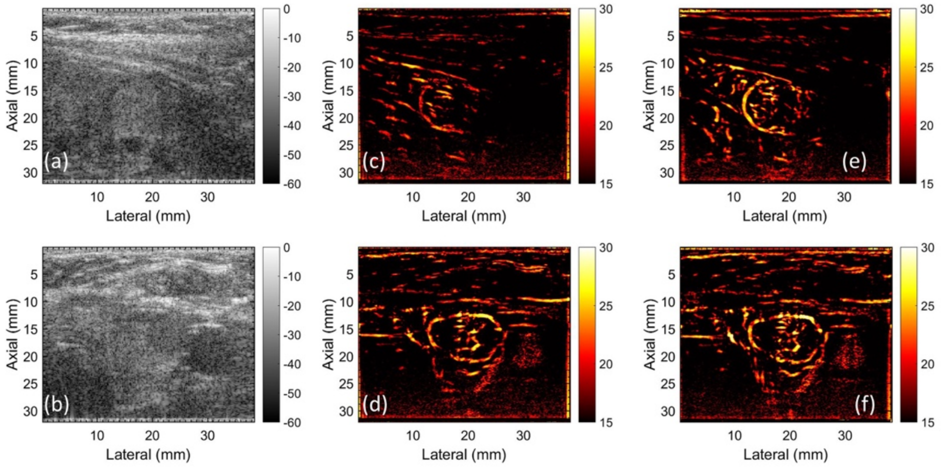

Fig. 10 displays the plane wave B-mode sonograms (a,b) and the corresponding PD images – without (c,d) and with (e,f) motion correction. (a,c,e) corresponds to the longitudinal plane, and (b,d,f) corresponds to transverse plane, for patient # 21.

Supplementary Data

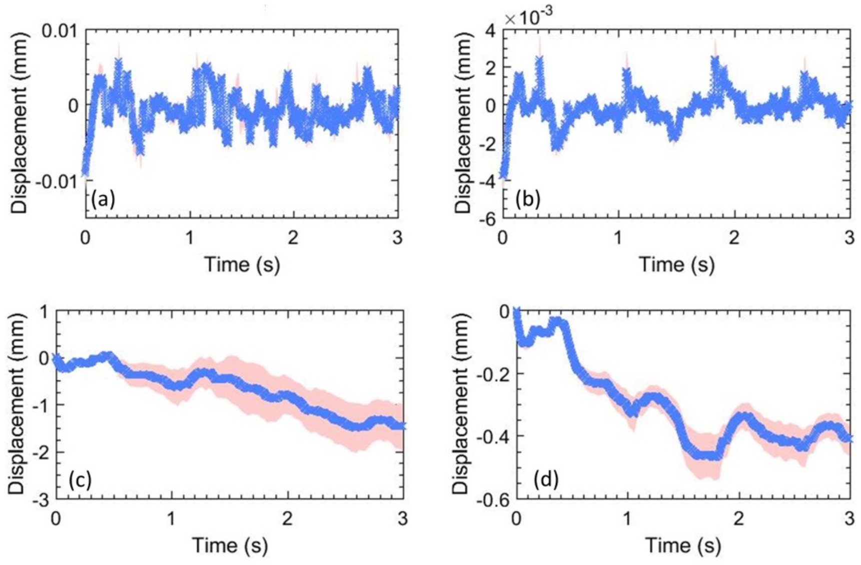

Fig. 11 (a,b) and (c,d) display the lateral and axial displacements, respectively, estimated in the longitudinal view of the thyroid for patient # 21. Specifically, (a,c) show the displacements estimated between consecutive frames, and (b,d) show the total accumulated displacements, with reference to the first frame in the ensemble. The continuous error-band (red) displays 1 standard deviation from the mean.

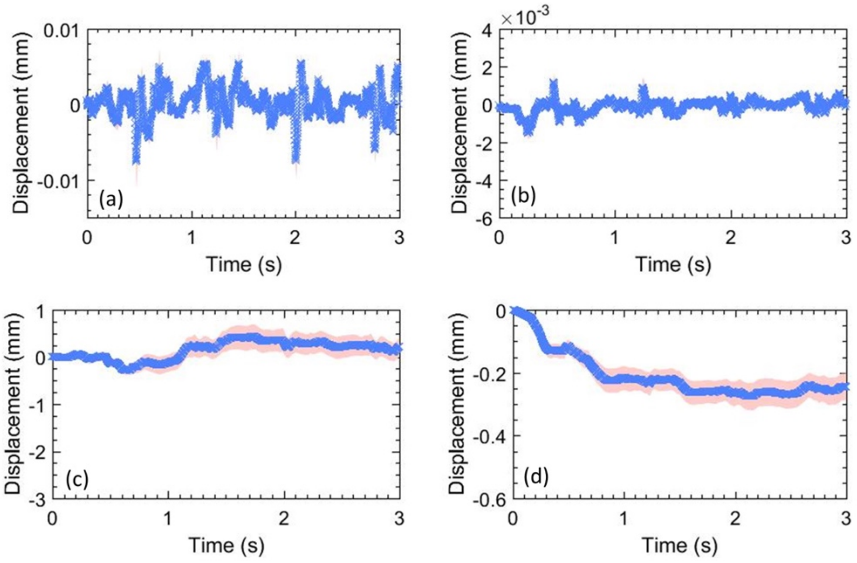

Fig. 12 (a,b) and (c,d) display the lateral and axial displacements, respectively, estimated in the transverse view of the thyroid for patient # 21. Specifically, (a,c) show the displacements estimated between consecutive frames, and (b,d) show the total accumulated displacements, with reference to the first frame in the ensemble. The continuous error-band (red) displays 1 standard deviation from the mean.

Supplementary Data

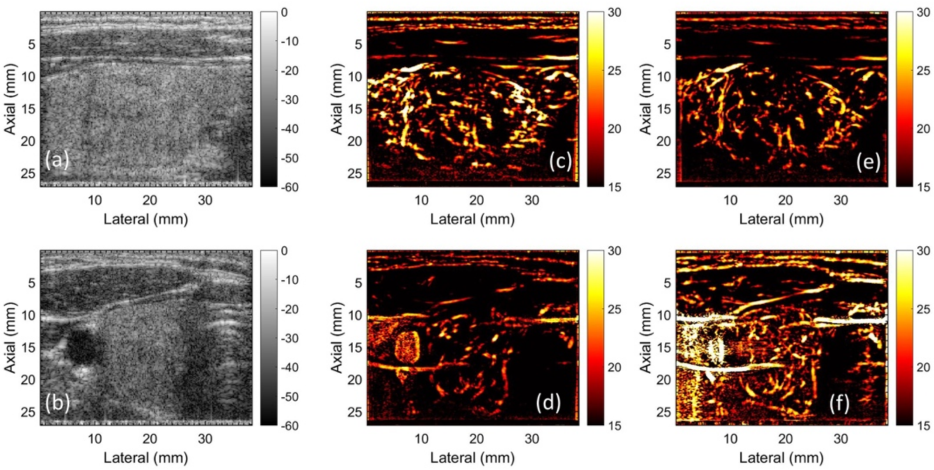

Fig. 13 displays the plane wave B-mode sonograms (a,b) and the corresponding PD images – without (c,d) and with (e,f) motion correction. (a,c,e) corresponds to the longitudinal plane, and (b,d,f) corresponds to transverse plane, for patient # 23.

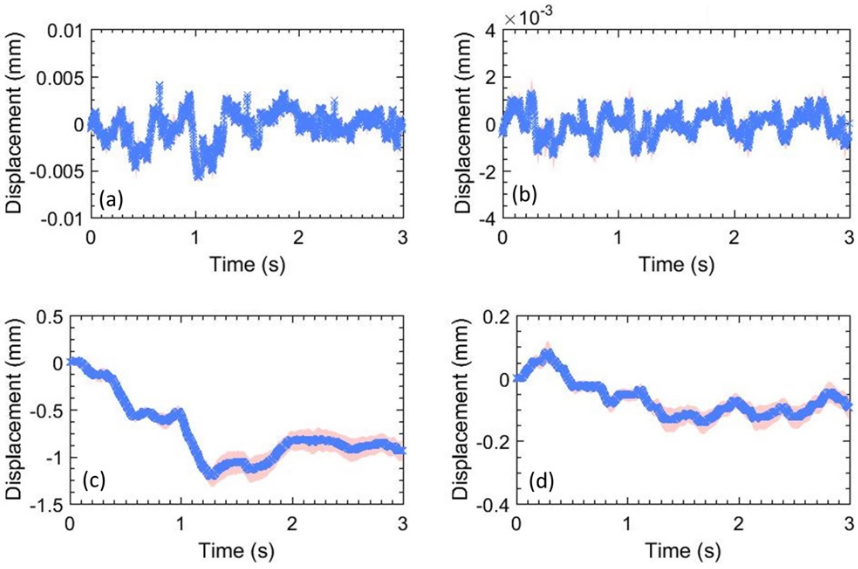

Fig. 14 (a,b) and (c,d) display the lateral and axial displacements, respectively, estimated in the longitudinal view of the thyroid for patient # 23. Specifically, (a,c) show the displacements estimated between consecutive frames, and (b,d) show the total accumulated displacements, with reference to the first frame in the ensemble. The continuous error-band (red) displays 1 standard deviation from the mean.

Supplementary Data

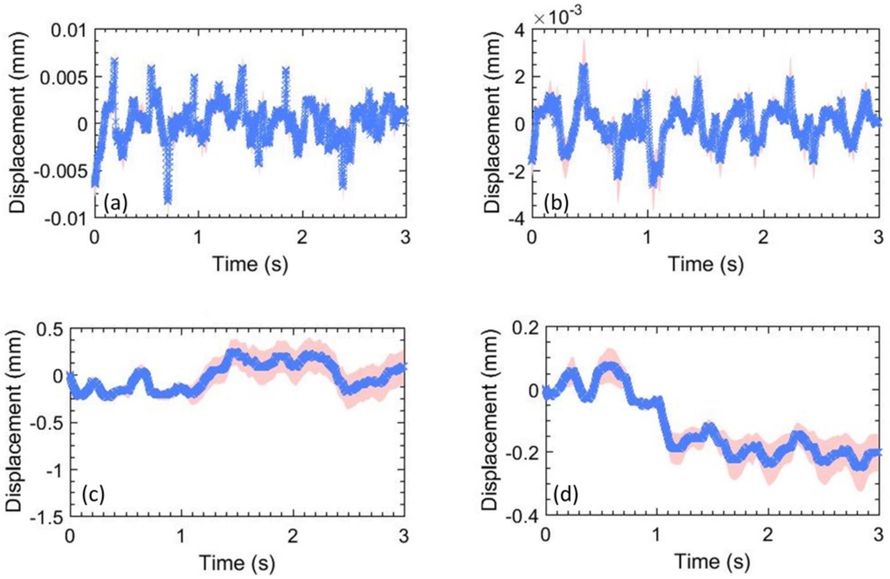

Fig. 15 (a,b) and (c,d) display the lateral and axial displacements, respectively, estimated in the transverse view of the thyroid for patient # 23. Specifically, (a,c) show the displacements estimated between consecutive frames, and (b,d) show the total accumulated displacements, with reference to the first frame in the ensemble. The continuous error-band (red) displays 1 standard deviation from the mean.

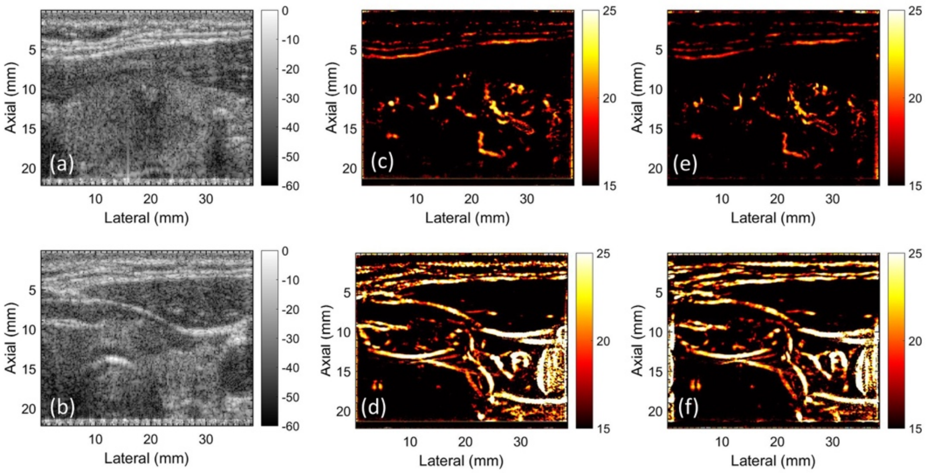

Fig. 16 displays the plane wave B-mode sonograms (a,b) and the corresponding PD images – without (c,d) and with (e,f) motion correction. (a,c,e) corresponds to the longitudinal plane, and (b,d,f) corresponds to transverse plane, for patient # 24.

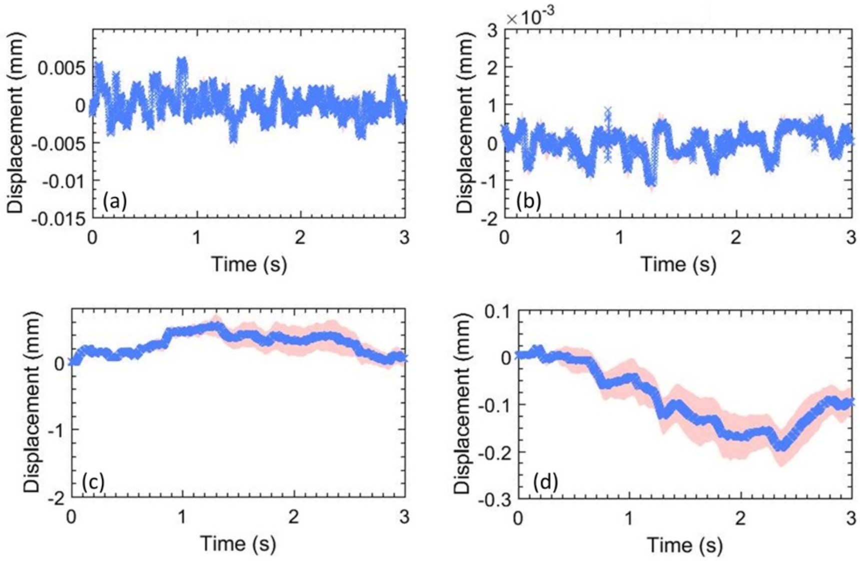

Fig. 17 (a,b) and (c,d) display the lateral and axial displacements, respectively, estimated in the longitudinal view of the thyroid for patient # 24. Specifically, (a,c) show the displacements estimated between consecutive frames, and (b,d) show the total accumulated displacements, with reference to the first frame in the ensemble. The continuous error-band (red) displays 1 standard deviation from the mean.

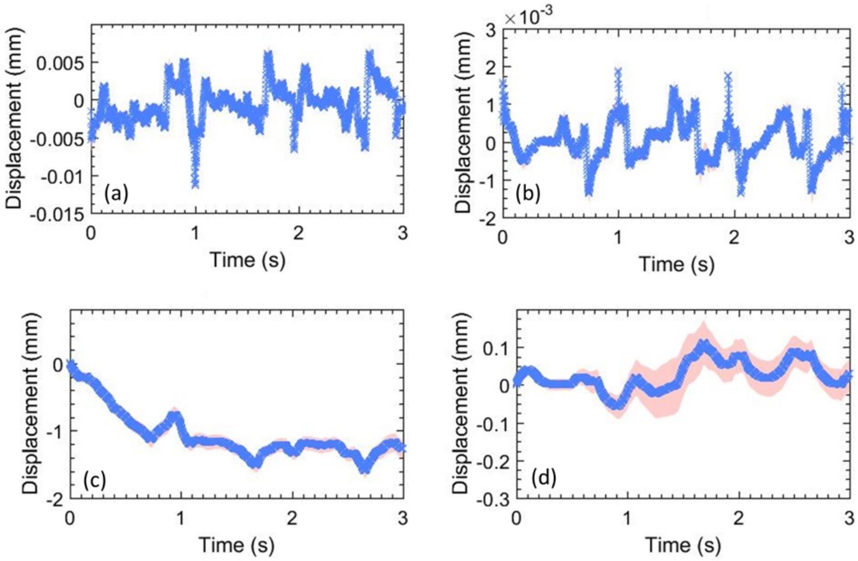

Fig. 18 (a,b) and (c,d) display the lateral and axial displacements, respectively, estimated in the transverse view of the thyroid for patient # 24. Specifically, (a,c) show the displacements estimated between consecutive frames, and (b,d) show the total accumulated displacements, with reference to the first frame in the ensemble. The continuous error-band (red) displays 1 standard deviation from the mean.

Supplementary Data

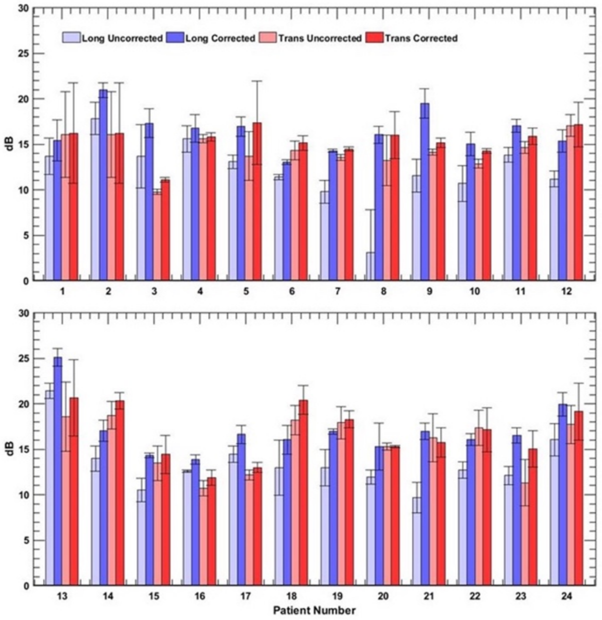

Fig. 19 displays barplot of CNR associated with the PD images of the 24 patients, with and without motion correction, in longitudinal and transverse views. The errorbars correspond to ±1 standard deviation.

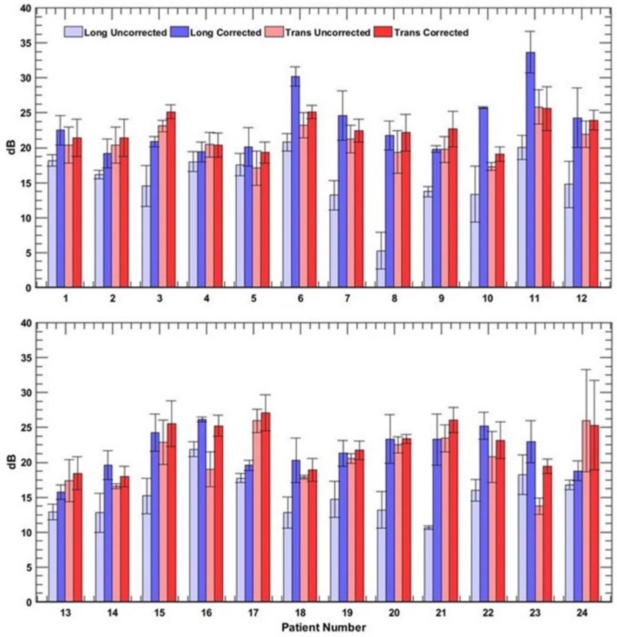

Fig. 20 displays barplot of SNR associated with the PD images of the 24 patients, with and without motion correction, in longitudinal and transverse views. The errorbars correspond to ±1 standard deviation.
